# Functional Profile Differences Across Diagnostic Categories Using WHODAS 2.0 in Adults with Neurological, Musculoskeletal, and Chronic Pain Conditions

**DOI:** 10.3390/jfmk10030312

**Published:** 2025-08-14

**Authors:** Patricio Barria, Asterio Andrade, Bessié Córdova Albayay, Felipe Covarrubias-Escudero, Carlos Cifuentes, Juan Camilo Moreno, Juan Pablo Appelgren-González

**Affiliations:** 1 Research and Development Area, Corporación de Rehabilitación Club de Leones Cruz del Sur, Punta Arenas 6210005, Chile; pbarria@rehabilitamos.org (P.B.); aandrade@rehabilitamos.org (A.A.); bessie.cordova@rehabilitamos.org (B.C.A.); 2 Translational Research Unit, Trainfes Center, Santiago 8760903, Chile; felipe@trainfes.com; 3 Department of Kinesiology, Faculty of Art and Physical Education, Universidad Metropolitana de Ciencias de la Educación, Santiago 7750332, Chile; 4 Bristol Robotics Laboratory, University of the West of England, Bristol BS16 1QY, UK; carlos.cifuentes@uwe.ac.uk; 5 Center for Automation and Robotics, UPM-CSIC, 28500 Madrid, Spain; jc.moreno@csic.es; 6 Center of Biomedical Imaging, Pontificia Universidad Católica de Chile, Santiago 7510000, Chile

**Keywords:** functional disability, WHODAS 2.0, chronic conditions, neurological disorders, musculoskeletal diseases, sex differences, pain-related conditions

## Abstract

**Background**: Functional disability is a growing concern in aging populations with chronic health conditions, yet few studies have compared disability profiles across diagnostic categories using standardized tools. **Objectives**: This study aimed to characterize the functional profiles of adults with neurological, musculoskeletal, and chronic pain conditions using the World Health Organization Disability Assessment Schedule 2.0 (WHODAS 2.0) and to examine differences by age and sex. **Methods**: A total of 419 participants (median age = 73 years; 73% female) completed the 36-item WHODAS 2.0. Diagnoses were classified into three groups: neurological (*n* = 134), musculoskeletal (*n* = 230), and pain-related (*n* = 55). Domain-level scores were analyzed using non-parametric tests and Spearman correlations. **Results**: revealed that neurological conditions were associated with the highest disability levels, particularly in cognition, interpersonal relations, and participation. Musculoskeletal conditions showed greater impairments in mobility and self-care, while pain-related conditions demonstrated variable disability, especially in participation. Women reported higher disability scores in the neurologic group, with significant differences observed in the cognition domain among neurological cases (*p* = 0.048). Age was positively correlated with disability in self-care and mobility, especially in musculoskeletal conditions. **Conclusions**: These findings highlight the utility of WHODAS 2.0 in identifying domain-specific limitations across clinical populations. They support the need for individualized, diagnosis- and gender-sensitive rehabilitation strategies, and suggest that WHODAS 2.0 can inform targeted care planning and resource allocation in rehabilitation settings. Future research should incorporate longitudinal designs and explore contextual factors influencing functional outcomes.

## 1. Introduction

Functional disability is an increasing global public health challenge due to population aging and the rising burden of chronic non-communicable diseases. It involves difficulties performing basic daily activities that reduce autonomy, social participation, and well-being [1]. An estimated 15% of adults worldwide are affected, with 2–4% experiencing severe impairments [2], marking a notable increase compared to earlier estimates from the 1970s, which reflects improved measurement methods and the growing prevalence of chronic diseases [3]. Recent data suggest that 12.6% of adults globally experience functional difficulties, with variability across countries due to differences in measurement tools [4]. These limitations relate not only to medical outcomes but also to social exclusion, reduced quality of life, and increased healthcare use [5]. Recognizing this, the World Health Organization has redefined healthy aging as maintaining functional capacity to support well-being in older age, emphasizing that functional impairments are not inevitable consequences of aging but modifiable when detected early [6]. Thus, functional disability remains a core indicator for equitable health system planning [7].

Prevalence rates are consistently higher among women, older adults, and socioeconomically disadvantaged populations [2]. Multiple studies have identified age as a key determinant, with prevalence rising sharply in older age groups. Disability prevalence rises with age (e.g., from 22.4% at 60–64 years to 54.8% after age 80 [8], and is higher among women, partly due to socioeconomic disparities and caregiving roles [9]. Rural populations and those with lower income/education face additional barriers [10], highlighting structural inequalities.

With rising life expectancy, strategies such as early detection and person-centered rehabilitation are essential [11]. A biopsychosocial approach helps move beyond descriptive trends, acknowledging the interplay of biological, psychological, and social factors [12]. For example, chronic pain reflects not only tissue damage but also coping and social support [13]. 

The WHO’s ICF framework promotes a biopsychosocial understanding of disability, shifting focus from disease-centric models to one where functionality emerges from interactions between health conditions and context [14]. It guides rehabilitation strategies that foster adaptive changes, supported by neuroplasticity [15].

Evidence suggests that robust social networks and a supportive environment can buffer the impact of disease on functional ability, even in older adults facing multiple health challenges [16]. Therefore, incorporating the concept of functional aging into rehabilitation planning encourages a preventive, person-centered model that accounts for both biological vulnerabilities and protective social factors [6].

To support person-centered care and enable standardized assessments of functioning beyond diagnosis, the WHO Disability Assessment Schedule 2.0 (WHODAS 2.0) has become a key instrument in rehabilitation. Grounded in the International Classification of Functioning, Disability and Health (ICF), WHODAS 2.0 reflects the biopsychosocial model through six core domains: cognition, mobility, self-care, getting along, life activities, and social participation [17,18]. Available in 36- and 12-item versions, and adaptable to self-, interviewer-, or proxy-report, it assesses functioning over the last 30 days using a five-point Likert scale and provides both simple and complex scoring systems that translate into a 0–100 scale, where higher scores reflect greater disability [17,18].

WHODAS 2.0 has demonstrated strong psychometric properties across neurological, musculoskeletal, and chronic pain conditions, including high internal consistency, test–retest reliability, and sensitivity to change [18,19]. Its construct validity has been confirmed in populations with Parkinson’s disease, multiple sclerosis, and stroke, as well as in musculoskeletal disorders like osteoarthritis and chronic back pain [17,18]. In chronic pain syndromes such as fibromyalgia, it captures the interplay between physical discomfort, emotional burden, and social isolation [20]. Despite this versatility, most studies have analyzed single-diagnosis cohorts and have not fully exploited the instrument’s potential for cross-diagnostic, multifactorial disability profiling [17,18], particularly regarding the interaction of diagnosis with age, sex, and socioeconomic vulnerability.

Despite the widespread clinical adoption of WHODAS 2.0, several limitations in the literature restrict its utility for population-level and cross-diagnostic analyses. Although the instrument was designed to allow comparisons across diagnostic categories, most empirical studies have focused on single-diagnosis cohorts—such as individuals with Parkinson’s disease, chronic back pain, or musculoskeletal conditions—limiting the generalizability of their findings [18]. Additionally, few studies have explored domain-specific functional profiles across multiple health conditions while accounting for key demographic variables such as age, sex, and socioeconomic vulnerability [17]. This gap constrains our understanding of how chronic conditions intersect with social determinants to shape disability trajectories.

Moreover, few studies have conducted comprehensive multivariate analyses to examine how functional performance is shaped by the interaction of diagnostic group, age, gender, and other contextual factors [18,21]. The lack of integrative analyses hinders the design of truly individualized rehabilitation strategies that reflect the complex reality of patients with multimorbidity and diverse sociodemographic profiles.

Age-related differences are particularly underexplored at the domain level. Evidence suggests that middle-aged adults (50–64 years) may already experience significant declines in certain domains of functioning, which tend to worsen with age [22]. Similarly, while women consistently report higher levels of disability, there is insufficient data to determine whether this is due to biological differences, social roles, or disparities in access to services [23]. Addressing these analytical gaps is essential for advancing equity and precision in functional rehabilitation planning.

In response, this study aims to generate new insights into how diagnosis, age, and sex interact to shape functional disability across multiple domains. Using WHODAS 2.0 as a standardized assessment tool, it seeks to characterize the domain-specific functional profiles of adults living with neurological, musculoskeletal, and chronic pain conditions, considering age, sex, and diagnosis as key explanatory variables.

By moving beyond single-disease analyses and adopting a comparative, multivariable approach, the study intends to provide a more nuanced understanding of the interaction between clinical and demographic factors in shaping disability outcomes. This information is crucial for informing targeted rehabilitation strategies, tailoring interventions to individual needs, and improving health service planning in complex and heterogeneous populations. Ultimately, the study contributes to the development of evidence-based, equitable, and person-centered care models within diverse healthcare systems.

## 2. Materials and Methods

### 2.1. Study Design

This research followed an observational cross-sectional design based on a secondary analysis of clinical data from a rehabilitation center registry in Chile. This design was chosen to describe functional disability profiles at a single point in time across different chronic condition groups.

### 2.2. Participants

Participants were drawn from the clinical registry of a rehabilitation network and represented three major diagnostic categories: neurological disorders, musculoskeletal diseases, and chronic pain-related syndromes.

Inclusion criteria required participants to (1) be aged 18 years or older, (2) have a confirmed diagnosis within one of the three predefined chronic condition categories, and (3) have completed the full WHODAS 2.0 questionnaire as part of their functional assessment process. Only patients with complete demographic and diagnostic records were retained for analysis.

Exclusion criteria included (1) incomplete clinical data, particularly absence of key variables such as age, sex, or diagnosis; and (2) severe cognitive or communicative impairments that precluded reliable self-report or caregiver-assisted completion of WHODAS 2.0.

Participant selection was conducted retrospectively from institutional databases, applying non-probabilistic sampling of all eligible individuals assessed between 2022 and 2024. This full sample approach was chosen to ensure representation across conditions and enhance external validity by maximizing sample size within each diagnostic category.

### 2.3. Outcome Measures

The primary outcome assessed in this study was functional disability, defined as the degree of difficulty experienced by individuals in performing activities of daily living and participating in community and social life. Given the multidimensional nature of disability, a comprehensive evaluation approach was required to capture limitations across cognitive, physical, interpersonal, and occupational domains.

This study utilized the 36-item version of World Health Organization Disability Assessment Schedule 2.0 (WHODAS 2.0), a standardized and validated instrument designed to measure functioning and disability independent of diagnosis and across diverse cultural contexts, across six core domains of functioning: cognition (understanding and communication), mobility (physical movement), self-care (personal hygiene, dressing, and eating), getting along with others (interpersonal interactions), life activities (domestic responsibilities and work/school engagement), and participation (involvement in society and community life).

Each item is rated on a 5-point Likert scale ranging from 0 (“no difficulty”) to 4 (“extreme difficulty or cannot do”). The raw scores for each domain are summed, resulting in both domain-specific scores and a total score. The maximum total score is 144 points, with higher values indicating greater disability. For this study, the simple scoring method was applied, which allows direct summation of responses without item weighting, facilitating interpretation and analysis across subgroups.

WHODAS 2.0 has demonstrated strong psychometric properties. According to WHO field trials and validation studies, the 36-item version shows high internal consistency (Cronbach’s alpha > 0.90 for the total score; >0.80 for domains) and excellent test–retest reliability (ICC = 0.93–0.98) [17,18]. It also exhibits good construct and concurrent validity across general and clinical populations with chronic neurological, musculoskeletal, and pain-related conditions. Its alignment with the ICF framework further enhances conceptual and clinical relevance.

The choice of WHODAS 2.0 was based on its methodological and practical advantages. First, it is a cross-culturally validated tool, suitable for use in diverse clinical settings, including rehabilitation centers and outpatient services. Second, it permits comparison across diagnostic groups by focusing on functioning rather than disease-specific symptoms. This makes it ideal for studies involving heterogeneous populations with chronic conditions. Third, the availability of a 36-item version provides granular data at the domain level, which aligns with the objective of this study to examine differential patterns of functional disability in detail. Lastly, the instrument’s widespread use in global disability research enhances the generalizability and comparability of findings.

### 2.4. Statistical Analysis

All statistical analyses were conducted using GraphPad Prism version 9.5.1 and PAST 4.12b. Descriptive statistics, inferential tests, and graphical visualizations were performed in alignment with data distribution characteristics. The dataset was first reviewed for completeness, duplicates, and consistency. Each domain and total score were extracted and categorized by sex, diagnosis, and age group for comparative analysis.

The Shapiro–Wilk test was applied to assess the normality of distribution for all WHODAS 2.0 domain scores and the total score. A non-parametric analytical approach was pre-specified given the ordinal nature and non-normal distribution of WHODAS 2.0 scores, justifying the use of non-parametric statistical methods for both descriptive and inferential analyses. Descriptive statistics were expressed as medians and interquartile ranges (IQRs) for each domain and for the total score, stratified by diagnostic group, sex, and age range. Categorical data (e.g., sex distribution across diagnoses) were reported as absolute frequencies and percentages.

For inferential analysis, group comparisons were conducted using the Mann–Whitney U test for two-group comparisons (e.g., male vs. female within a diagnostic group) and the Kruskal–Wallis test with Dunn’s post hoc correction for multiple comparisons across more than two groups. Spearman’s rank correlation coefficient was used to explore associations between age and WHODAS domain scores, both at group level and within specific diagnoses (e.g., osteoarthritis, fibromyalgia).

The threshold for statistical significance was established at α = 0.05. All *p*-values are reported explicitly, and where multiple comparisons were performed, adjusted *p*-values were applied using Dunn’s method. Box plots and radar plots were generated to depict central tendency and variation across domains, diagnostic categories, and sex. Linear regression trend lines were plotted in cases where age was explored as a predictor of functional domain scores.

### 2.5. Ethical Considerations

This study was conducted in accordance with international ethical standards for research involving human subjects. The research protocol was reviewed and approved by the Institutional Ethics Committee of the Rehabilitation Center Club de Leones Cruz del Sur, under approval code CRCS_UID_010223, 1 February 2023, ensuring compliance with institutional and national guidelines for biomedical research.

All procedures followed the ethical principles outlined in the Declaration of Helsinki (last revision, 2013) and adhered to the guidelines issued by the Council for International Organizations of Medical Sciences (CIOMS) for the ethical conduct of research involving vulnerable populations.

Written informed consent was obtained from all participants prior to inclusion in the study. In cases where participants were unable to provide consent directly due to health or communication limitations, authorization was obtained from a legally authorized representative, in accordance with ethical protocols.

To ensure data confidentiality and participant privacy, all datasets were anonymized before analysis. Personal identifiers were removed, and each participant was assigned a coded identifier. Access to sensitive information was restricted to authorized personnel involved in the data handling and analysis process.

Participation in the study was entirely voluntary, and no financial compensation or material incentive was provided to participants. There were no foreseeable risks associated with participation, as all procedures were based on routine clinical assessments and secondary analysis of pre-existing data.

## 3. Results

### 3.1. Sample Characteristics and Overall WHODAS Scores

A total of 423 participants completed the WHODAS 2.0 assessment, of whom 419 were included in the final analysis after excluding four cases due to missing clinical data. The participants’ median age was 73 years (IQR: 64–80), and 306 (73%) were female, as shown in Table 1. Diagnoses were categorized into three groups: musculoskeletal (*n* = 230), neurological (*n* = 134), and pain-related conditions (*n* = 55). The highest median domain scores across the full sample were observed in mobility (D2: 2.40), life activities (D5: 2.00), and participation (D6: 1.88), while the lowest were in interpersonal relationships (D4: 0.80) and self-care (D3: 1.25). The total simple score had a median of 52.00 (IQR: 34.00–69.00). Shapiro–Wilk tests confirmed non-normal distributions for all variables (*p* < 0.05), supporting the use of non-parametric analyses.

### 3.2. Disability Profiles by Diagnostic Group

Median total scores varied across diagnostic categories, with the highest disability observed in neurological conditions (median = 59.00), followed by pain-related conditions (49.00) and musculoskeletal disorders (48.00) (Table 2).

Domain-specific differences were also evident:

- Neurological conditions showed the greatest overall disability, with the highest scores in mobility (D2: 2.50), life activities (D5: 2.06), and participation (D6: 2.00). Cognition (D1: 1.33) and self-care (D3: 1.75) were also notably affected.
- Musculoskeletal disorders presented prominent difficulties in mobility (D2: 2.20) and life activities (D5: 1.75), with milder impairments in cognition (D1: 1.00) and participation (D6: 1.75).
- Pain-related conditions had a more heterogeneous profile, with comparable scores in mobility (D2: 2.20) and participation (D6: 2.00), but lower disability in self-care (D3: 0.75) and cognition (D1: 1.17).

### 3.3. Neurological Subgroup Analysis

**Figure 1:**
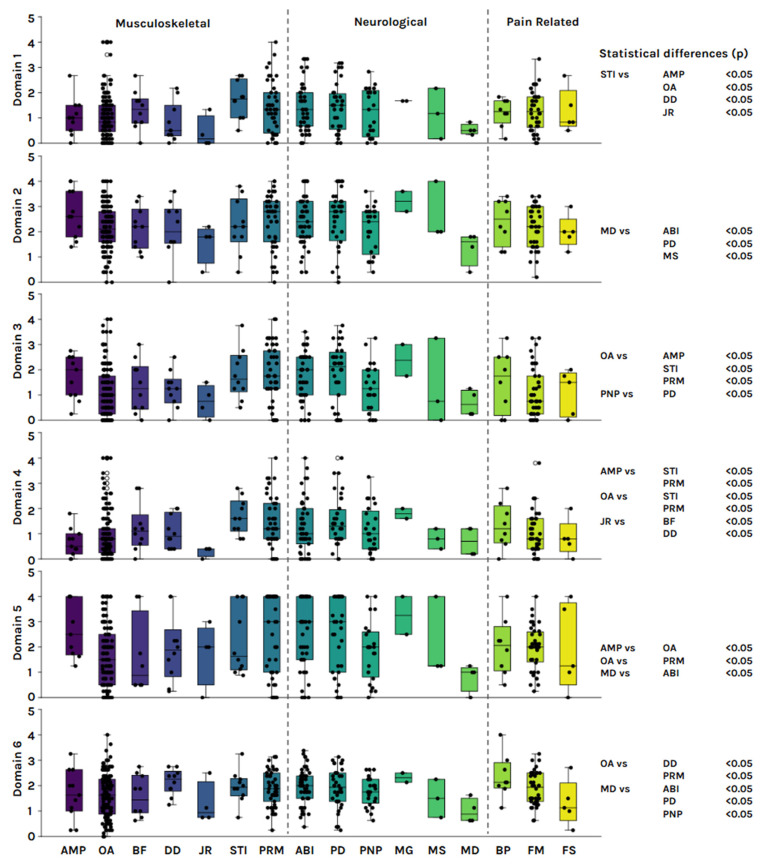
Subcategory distribution of WHODAS 2.0 scores across all six domains. Mann–Whitney’s *p* value between subcategories expressed on the right side of the figure. AMP: amputee; OA: osteoarthritis; BF: bone fracture; DD: disc disease; JR: joint replacement; STI: soft tissue injuries; PRM: people with reduced mobility; ABI: acquire brain injury; PD: Parkinson’s disease; PNP: polyneuropathy; MG: Miastenia Gravis; MS: multiple sclerosis; MD: muscular dystrophy; FM: fibromyalgia; FS: frozen shoulder.

Within the neurological group (*n* = 134), the most frequent diagnoses were acquired brain injury (*n* = 47), Parkinson’s disease (*n* = 36), and polyneuropathy (*n* = 25). Across all domains, female participants consistently reported higher levels of disability than males. The median total simple score was 62.5 for women and 51.5 for men. A statistically significant sex difference was observed in the cognition domain (D1; *p* = 0.048). Detailed median domain scores by diagnosis and sex are presented in Table 2 and illustrated in Figure 1.

#### 3.3.1. Acquired Brain Injury

Among the 47 individuals with acquired brain injury (ABI), 30 were female and 17 were male. The most pronounced intersex difference was observed in the life activities domain (D5), with a median score of 3.00 for women and 1.75 for men. While women showed higher scores across all WHODAS 2.0 domains, no statistically significant sex differences were identified in this subgroup.

#### 3.3.2. Parkinson Disease

Among the 36 participants diagnosed with Parkinson’s disease (PD), 22 were women and 14 were men. The female subgroup presented a substantially higher total WHODAS 2.0 score (median = 68.00) compared to the male subgroup (median = 43.00). Although women exhibited consistently higher median scores across all domains, none of the sex-based differences reached statistical significance.

#### 3.3.3. Polyneuropathies

In the polyneuropathy (PNP) subgroup (*n* = 25), 18 participants were female and 7 were male. A statistically significant sex difference was observed in the cognition domain (D1), with women reporting a median score of 2.00 compared to 0.17 in men (*p* = 0.007). Although women also exhibited higher scores in other domains, none of those differences were statistically significant.

### 3.4. Musculoskeletal Subgroup Analysis

The musculoskeletal group included 230 participants, of whom 171 were female and 59 were male. The median total WHODAS 2.0 score was 48.00 in women and 53.00 in men, a difference that did not reach statistical significance (*p* = 0.068). Detailed median scores by diagnosis and sex are presented in Table 2.

A multivariable correlation analysis revealed positive associations between age and several WHODAS domains, particularly in cognition (D1), mobility (D2), self-care (D3), and getting along (D4). The strongest correlation was observed between age and the self-care domain (D3), with a Spearman coefficient of ρ = 0.21 (*p* < 0.001).

When stratifying by diagnosis, this association was even more pronounced in participants with osteoarthritis (*n* = 130), where the correlation between age and self-care reached ρ = 0.25 (*p* < 0.001). No significant differences were observed between sexes in this correlation.

Domain-level scores varied across the different musculoskeletal diagnoses. Among People with Reduced Mobility (PRM, *n* = 47), higher disability scores were observed in mobility (D2), life activities (D5), and participation (D6). Individuals with amputation (*n* = 11) also showed elevated scores, particularly in self-care (D3) and life activities (D5). Lower levels of disability were reported in conditions such as joint replacement and bone fracture. Table 2 provides a detailed breakdown of domain scores and total scores by sex for each musculoskeletal diagnosis.

### 3.5. Pain-Related Subgroup Analysis

The pain-related group comprised 55 participants, including 42 individuals with fibromyalgia, 8 with back pain, and 5 with painful shoulder syndrome. Only three male participants were included in this group (one per diagnostic category), preventing meaningful statistical comparisons by sex.

The median total WHODAS 2.0 score for the group was 49.00. Domain-level scores showed functional variability across diagnoses, with the highest scores observed in participation (D6) and mobility (D2). Fibromyalgia was the most prevalent condition and demonstrated consistently moderate-to-high disability across all domains, whereas painful shoulder syndrome showed comparatively lower scores.

A multivariable correlation analysis revealed a significant negative association between age and participation (D6), with a Spearman coefficient of ρ = −0.35 (*p* < 0.001). This indicates that older participants reported lower disability in participation-related tasks. A similar trend was observed for the total WHODAS score, although it did not reach statistical significance. These findings may reflect the adoption of age-related coping strategies or lower perceived role expectations among older adults. Table 2 summarizes the median scores for each domain and the total score across the three diagnostic categories.

## 4. Discussion

This study aimed to characterize the functional profiles of adults with neurological, musculoskeletal, and chronic pain conditions using WHODAS 2.0, with special attention to differences by diagnosis, age, and sex. The results revealed that disability profiles differ substantially between clinical groups, with neurological conditions presenting the greatest overall disability, particularly in the domains of cognition, mobility, and participation. Musculoskeletal diseases showed greater limitations in mobility and self-care, while chronic pain conditions presented a more heterogeneous distribution, with a special impact on social participation. Higher levels of disability were also observed in women with neurological conditions and a positive correlation between age and functional impairment particularly in people with musculoskeletal diseases.

The analysis by functional domains revealed differentiated patterns according to diagnosis, which reflect both the pathophysiological impact of each condition and contextual and adaptive factors. In neurological conditions, higher scores in cognition, participation, and mobility are consistent with central nervous system impairments and progressive loss of executive skills, social interaction, and physical displacement, as documented in patients with Parkinson’s or acquired brain injuries [18,24]. In the musculoskeletal group, the limitations were concentrated in mobility and self-care, which can be explained by the presence of pain, joint stiffness, or cumulative motor impairment, especially in older adults with osteoarthritis. On the other hand, chronic pain conditions such as fibromyalgia showed a more variable profile, but with a notable affectation in participation, which could be linked to behavioral avoidance processes, fatigue, and emotional alterations [25]. In the musculoskeletal group, the limitations were concentrated in mobility and self-care, which can be explained by the presence of pain, joint stiffness, or cumulative motor impairment, especially in older adults with osteoarthritis. On the other hand, chronic pain conditions such as fibromyalgia showed a more variable profile, but with a notable affectation in participation, which could be linked to behavioral avoidance processes, fatigue, and emotional alterations.

The functional differences according to sex and age observed in this study reinforce the importance of considering sociodemographic variables in rehabilitation planning. Women reported higher levels of disability in almost all domains, with statistically significant differences in the cognitive area within the neurological group. These findings are consistent with the literature that attributes these differences not only to biological factors, but also to the cumulative weight of structural inequalities, such as less access to specialized services, burden of caregiving roles, and greater exposure to chronic stress [9,26]. The functional differences according to sex and age observed in this study reinforce the importance of considering sociodemographic variables in rehabilitation planning. Women reported higher levels of disability in almost all domains, with statistically significant differences in the cognitive area within the neurological group. These findings are consistent with the literature that attributes these differences not only to biological factors, but also to the cumulative weight of structural inequalities, such as less access to specialized services, burden of caregiving roles, and greater exposure to chronic stress [11]. These associations reinforce the need to apply a life-course perspective in rehabilitation, anticipating functional impairment and promoting preventive strategies that consider both individual trajectories and social determinants of health.

From a clinical and programmatic perspective, the results of this study underscore the need to design differentiated interventions according to diagnosis, age, and gender. In people with neurological conditions, the simultaneous involvement of cognitive, motor, and participation domains suggests that rehabilitation programs should integrate physical and neurocognitive components, as well as strategies for social reintegration [18,27]. From a clinical and programmatic perspective, the results of this study underscore the need to design differentiated interventions according to diagnosis, age, and gender. In people with neurological conditions, the simultaneous involvement of cognitive, motor, and participation domains suggests that rehabilitation programs should integrate physical and neurocognitive components, as well as strategies for social reintegration [24,28]. For its part, in people with chronic pain, such as fibromyalgia, it is recommended to incorporate interdisciplinary interventions that address not only pain, but also emotional coping, fatigue, and functional self-perception [25]. In turn, the differences observed between women and men reinforce the need for gender-sensitive approaches, which consider inequalities in access to treatment, care responsibilities, and the stigma associated with certain conditions [9,26]. In turn, the differences observed between women and men reinforce the need for gender-sensitive approaches, which consider inequalities in access to treatment, care responsibilities, and the stigma associated with certain conditions [17,19].

The observed patterns can be interpreted through established theoretical frameworks. The biopsychosocial model supports the idea that disability arises from the interaction of physiological impairments, psychological resilience, and social–environmental context [12,14]. In neurological conditions, persistent impairments may reflect limited neuroplasticity or delayed access to specialized rehabilitation services [27]. Among older adults with musculoskeletal disorders, cumulative aging processes and limited mobility could lead to progressive loss of autonomy and increased participation restrictions, particularly in under-resourced contexts.

This study has certain limitations that must be considered when interpreting the results. First, the cross-sectional design prevents establishing causal relationships between the sociodemographic variables and the levels of functional disability observed. Second, the use of a single self-reporting tool such as WHODAS 2.0 could have introduced perception biases, particularly in people with cognitive impairments or low educational attainment, which could affect the accuracy of scores in some domains [17]. In addition, the study did not incorporate relevant contextual variables such as socioeconomic level, support networks, level of physical activity, or presence of comorbidities, which can significantly influence functionality, especially in older people [11]. Finally, although there was a diverse sample, the subgroup sizes were not homogeneous, which could limit the generalizability of the comparative analyses.

Future research should prioritize longitudinal cohort designs and robust multivariate analyses that incorporate contextual variables to better understand the complexity of functional disability across diverse populations. The integration of mixed methods—combining objective assessments (e.g., gait speed, grip strength, neuropsychological testing) with qualitative evaluations—would allow for a more comprehensive understanding of individual experiences and environmental influences. Additionally, the inclusion of social determinants such as income, housing, and social support would enhance the explanatory power of these studies. Advanced analytical strategies, including machine learning models, may further contribute by identifying predictive profiles of functional risk or recovery trajectories across various chronic health conditions.

Overall, the findings of this study reinforce the need to understand functional disability as a dynamic and multidimensional phenomenon, which requires differentiated approaches according to diagnosis, age, gender, and social context. Functional assessment through standardized tools such as WHODAS 2.0 makes it possible to identify critical domains of intervention, facilitating the design of more precise, equitable, and person-centered rehabilitation strategies. Adopting a view based on functional trajectories, beyond clinical diagnoses, represents a key step towards more inclusive and responsive health systems to human diversity. In this sense, the present study contributes to expanding the evidence base necessary to move towards personalized care models that promote autonomy, participation, and quality of life for people living with chronic conditions.

## 5. Conclusions

This study provides evidence that functional disability in adults with chronic neurological, musculoskeletal, and pain-related conditions varies meaningfully across diagnostic groups, sex, and age. The use of WHODAS 2.0 enabled a multidimensional understanding of how these populations experience limitations in daily functioning, with neurological conditions and female sex being consistently associated with greater impairment.

These findings contribute to the field by reinforcing the utility of domain-based assessment tools in rehabilitation settings and highlighting the need for more tailored, person-centered approaches to functional evaluation and treatment planning. The analysis supports the relevance of incorporating demographic and diagnostic stratification into clinical decision-making processes.

For the populations affected, the results underscore the importance of accessible, individualized rehabilitation strategies that address both physical and cognitive barriers to autonomy. The study also supports the integration of gender-sensitive perspectives in rehabilitation programs to reduce disparities in functional outcomes.

Future research should build on these findings by employing longitudinal designs, incorporating objective functional metrics, and exploring contextual factors such as social support and access to care. This will allow for more predictive, equitable, and effective rehabilitation interventions across diverse patient populations.

## Figures and Tables

**Table 1:** Demographic characteristics for each subgroup of conditions. Age represented in means and standard deviation. Sex, represented with the number of female patients and the percentage of the subcategory.

SUBCATEGORY	N	Age	Sex (F, %)
MSK TOTAL	230	74.8 ± 12.6	
Osteoarthritis (OA)	130	76 ± 10.4	109 (83.8)
People with Reduced Mobility (PRM)	47	77.4 ± 14.6	26 (55.3)
Amputation (AMP)	11	66.5 ± 10.3	6 (54.5)
Bone Fracture (BF)	10	80.1 ± 14.9	8 (80)
Disc Disease (DD)	10	58.7 ± 15.7	10 (100)
Soft Tissue Injury (STI)	10	68.9 ± 13.5	4 (40)
Joint Replacement (JR)	4	70 ± 11.7	3 (75)
Other Msk	8	74.1 ± 10.3	5 (62.5)
NEURO TOTAL	134	70 ± 14.8	
Acquire Brain Damage (ABI)	47	72.8 ± 13.1	30 (63.8)
Parkinson’s Disease (PD)	36	76.3 ± 8.3	22 (61.1)
Polyneuropathy (PNP)	25	69.8 ± 15.7	18 (72)
Muscular Dystrophy (MD)	4	42.3 ± 9.6	4 (100)
Multiple Sclerosis (MS)	3	59 ± 8	2 (66.7)
Miastenia Gravis (MG)	2	65 ± 15.6	2 (100)
Other Neuro	17	58.4 ± 22.8	10 (58.8)
PAIN TOTAL	55	59.5 ± 13.9	
Fibromyalgia (FM)	42	56.7 ± 12	41 (97.6)
Back Pain (BP)	8	60.9 ± 16.1	7 (87.5)
Frozen Shoulder (FS)	5	81 ± 6.4	4 (80)
TOTAL	419	71.2 ± 14.4	

**Table 2:** WHODAS 2.0 scores by sex and total across all subcategories.

SUBCATEGORY	GENDER	N	D1	D2	D3	D4	D5T	D6	Total
Acquire Brain Damage (Abi)	F	30	1.33	2.60	2.00	0.90	3.00	2.25	65.50
	M	17	1.17	2.20	1.50	1.20	1.75	2.00	51.00
	Total	47	1.33	2.40	2.00	1.00	3.00	2.00	61.00
Parkinson’s Disease (PD)	F	22	1.58	3.00	2.25	1.40	3.00	2.00	68.00
	M	14	0.67	1.90	1.75	0.80	2.25	1.38	43.00
	Total	36	1.50	2.80	2.13	1.30	3.00	1.94	66.00
Polyneuropathy (PNP)	F	18	2.00	2.20	1.38	1.00	2.00	1.88	58.13
	M	7	0.17	2.60	1.00	0.40	2.00	1.75	52.00
	Total	25	1.33	2.40	1.25	1.00	2.00	1.75	55.00
Miastenia Gravis (MG)	F	2	1.67	3.20	2.38	1.80	3.25	2.31	76.00
Multiple Sclerosis (MS)	F	2	0.67	2.00	0.38	0.60	1.25	1.13	35.00
	M	1	2.17	4.00	3.25	1.20	4.00	2.25	86.00
	Total	3	1.17	2.00	0.75	0.80	1.25	1.50	46.00
Muscular Dystrophy (MD)	F	4	0.50	1.60	0.63	0.70	1.00	0.88	29.00
Other Neuro	F	10	1.42	2.60	1.38	1.30	1.75	2.19	59.00
	M	7	1.17	1.60	1.50	0.80	2.00	2.00	59.00
	Total	17	1.17	2.40	1.50	1.00	2.00	2.13	59.00
All NEUROLOGICAL	Total	134	1.33	2.50	1.75	1.10	2.06	2.00	59.00
Amputation (AMP)	F	6	0.67	2.70	1.88	0.65	3.00	2.13	58.00
	M	5	1.17	1.80	2.00	0.40	1.88	1.43	45.43
	Total	11	1.00	2.60	2.00	0.50	2.50	1.63	54.00
Osteoarthritis (OA)	F	109	0.83	2.20	1.00	0.80	1.75	1.63	43.00
	M	21	1.00	2.00	0.75	0.80	1.00	1.50	37.00
	Total	130	0.83	2.10	1.00	0.80	1.50	1.63	41.50
Bone Fracture (BF)	F	8	1.00	1.90	0.75	0.90	0.50	1.00	33.50
	M	2	1.67	3.30	2.50	2.80	4.00	2.63	87.50
	Total	10	1.33	2.20	1.25	1.10	0.88	1.44	43.00
Disc Disease (DD)	F	10	0.50	2.00	1.25	0.90	1.88	2.25	53.50
Joint Replacement (JR)	F	3	0.00	1.80	1.00	0.40	2.00	1.13	34.00
	M	1	0.33	0.40	0.50	0.00	0.00	0.75	12.00
	Total	4	0.17	1.80	0.75	0.40	2.00	0.94	32.50
Soft Tissue Injury (STI)	F	4	1.83	2.20	1.50	1.80	1.13	2.06	60.00
	M	6	1.33	2.10	1.88	1.20	2.88	1.88	60.50
	Total	10	1.75	2.20	1.63	1.60	1.63	1.94	60.00
People with Reduced Mobility (PRM)	F	26	1.17	2.60	1.75	0.90	3.00	1.69	56.00
	M	21	1.50	3.00	2.00	1.60	4.00	2.13	72.00
	Total	47	1.33	2.80	1.75	1.20	3.00	1.88	61.00
Other MSK	F	5	0.67	2.00	0.00	0.00	1.25	1.00	35.00
	M	3	2.00	2.80	2.25	1.00	2.63	2.25	59.00
	Total	8	0.75	2.00	0.38	0.80	1.25	1.81	39.00
All MSK	Total	230	1.00	2.20	1.25	0.80	1.75	1.75	48.00
Back Pain (BP)	Total	8	1.25	2.50	1.75	1.20	2.06	2.13	57.00
Fibromyalgia (FM)	Total	42	1.25	2.20	0.75	0.80	2.00	1.94	48.00
Painful Shoulder (FS)	Total	5	0.83	2.00	1.50	0.80	1.25	1.13	35.00

## Data Availability

The data that support the findings of this study are available from the corresponding author upon reasonable request. Due to institutional policy and ethical restrictions, the datasets are not publicly available in order to protect patient confidentiality.
